# Influence of inoculation dose and route on EHDV-8 distribution and the induced immune response in experimentally infected cattle

**DOI:** 10.1186/s13567-025-01652-3

**Published:** 2025-11-21

**Authors:** Ilse De Leeuw, Ruben Villalba, Montserrat Aguëro, Laurent Mostin, Nick De Regge

**Affiliations:** 1 Infectious Diseases in Animals, Exotic and Vector-Borne Diseases, Sciensano, Groeselenberg 99, 1180 Brussels, Belgium; 2 Faculty of Veterinary Medicine, Department of Translational Physiology, Infectiology and Public Health, Ghent University, Salisburylaan 133, 9820 Merelbeke, Belgium; 3 Laboratorio Central de Veterinaria, Ministry of Agriculture, Fisheries and Food, 28110 Algete, Spain; 4 Experimental Center Machelen, Sciensano, Kerklaan 68, 1830 Machelen, Belgium

**Keywords:** EHDV-8, infection study, cattle, virus distribution, induced immune response

## Abstract

Epizootic haemorrhagic disease virus serotype 8 (EHDV-8) is an emerging *Culicoides*-borne virus that was first detected in Europe in autumn 2022 in Italy and subsequently spread to Spain, Portugal and France. Despite its economic impact, little is known about its tissue distribution, persistence, and induction of immune responses in cattle. Therefore, we conducted an experimental infection study in groups of 3 cattle inoculated intradermally (ID; 10^6.2^ or 10^5.2^ TCID_50_/animal) or subcutaneously (SC, 10^6.2^ TCID_50_/animal) with a Spanish EHDV-8 isolate. Viremia appeared at 3 days post-inoculation (dpi), peaked at 7–10 dpi, and persisted until the end of the study (21 dpi). Fever spiked at 8–9 dpi with a longer duration in the high-dose groups. No other clinical symptoms were noted, except in one animal from the SC high-dose group. This animal exhibited apathy, conjunctivitis, and swollen lymph nodes from 10 dpi onwards. At necropsy, lymph nodes and spleen contained higher viral loads than muscles, brain, and skin. The clinical animal displayed petechiae on the kidney with high viral loads (Ct value = 20), which was an outlier compared with all other organs tested. The virological data thus confirmed that blood, spleen and lymph nodes are the most suitable diagnostic samples. A strong humoral response (100% seroconversion) was observed in all the groups at 10 dpi. All the animals, except the one showing clinical signs, also exhibited a strong cellular immune response, with IFN-γ levels peaking at 5–7 dpi. In conclusion, this study demonstrated that the inoculation route and dose only minimally impacted viremia, viral spread and induced immune responses.

**Supplementary Information:**

The online version contains supplementary material available at 10.1186/s13567-025-01652-3.

## Introduction

Epizootic haemorrhagic disease (EHD) is a vector-borne viral disease that affects domesticated and wild ruminants [1]. The virus is transmitted via bites of *Culicoides* midges [2]. It is classified within the genus *Orbivirus* of the family *Sedoreoviridae* [3]. The EHD virus (EHDV) genome consists of 10 dsRNA segments (S1-S10) that encode 7 structural proteins (VP1-VP7) and 4 nonstructural proteins [4], with seven distinct serotypes currently identified, namely, 1, 2, and 4 to 8 [5].

EHDV was first identified in 1955 in New Jersey. It is recognized primarily for affecting white-tailed deer in North America, causing high mortality levels in this species [6]. In contrast, EHDV infections in cattle were historically considered asymptomatic, except for the EHDV-2 Ibaraki strain [7–9]. Notable changes have occurred in the geographical distribution of EHDV. While this virus has been reported in North America, Africa, Australia, and Asia [8], outbreaks of EHDV-1, −6 and −7 have been reported in countries near Europe (e.g., Turkey, Israel, Tunisia) in the last 2 decades. These countries were previously considered free regions [10–12]. Simultaneously, the clinical presentation in cattle has shifted as well, especially for EHDV-6 and EHDV-7, from predominantly subclinical to clinical disease. Clinical signs include fever, hyperaemia, oral ulcers, excessive salivation, increased nasal and ocular secretions, lethargy, weakness, lameness, loss of appetite and conjunctivitis, with occasional fatalities reported [1, 10, 13]. Owing to these changes and significant economic implications, EHDV was added to the WOAH list of notifiable diseases in 2008 [14].

In 2022, EHDV was detected in Europe for the first time, with outbreaks reported in Italy (Sardinia and Sicily) and southern Spain, affecting cattle, deer, and sheep [15, 16]. The virus, identified as serotype 8, has shown more than 99% genetic similarity to the EHDV-8 strain that has circulated in Tunisia since 2021 [15]. The virus overwintered successfully in Europe, maintaining its circulation until 2023. By the summer of 2023, it had spread throughout Spain, reached Portugal, and by September, extended into France, infecting more than 4000 herds across multiple departments [17]. In the summer of 2024, new outbreaks were detected in Spain, Portugal and France [18]. Field observations in cattle revealed clinical signs such as respiratory distress, muzzle and oral mucosa erosions, drooling, inappetence, cyanosis and oedema of the tongue, conjunctivitis, fever, recumbency, coronary band injuries and, in severe cases, death [15, 19]. However, data on clinical signs in the literature remain limited.

Knowledge about EHDV-8 is even more limited than that of other EHDV serotypes, as this serotype had previously only been reported in Australia in 1982 [14]. Significant genomic differences exist between the circulating Mediterranean EHDV-8 strain and the strain from Australia. Segment 2, encoding an outer capsid protein that determines the serotype, displays only 77% homology at the nucleotide level [20]. Furthermore, phylogenetic analysis revealed that the other 9 segments from the Australian EDHV-8 cluster differently than those from the Mediterranean strain [17]. Therefore, available tests and scientific knowledge about EHDV-8 cannot be extrapolated as such. This is nicely demonstrated by the discrepancy in clinical outcomes following an EHDV-8 infection study carried out in Australia [21] and Italy [22]. The Australian study revealed clinical signs in sheep but not in cattle, whereas the Italian study reported fever and nasal lesions in cattle, fever in sheep, and no clinical signs in goats.

The European livestock sector faces new challenges with the introduction of EHDV, as restrictions are imposed on the trade of live ruminants, such as cattle, sheep and goats, from affected regions [Commission Delegated Regulation (EU) 2020/688 of 17 December 2019]. However, two EHDV-8 vaccines, an inactivated and a subunit differentiating infected from vaccinated animals (DIVA) vaccine, have been recently approved for use despite not having formal market authorization [Commission Delegated Regulation (EU) 2019/6 (Article 110)].

To address some of the existing knowledge gaps concerning the interaction between the newly emerged EHDV-8 strain and its hosts, this study examined EHDV-8 tissue distribution, persistence and induced immune responses in cattle. In addition, we evaluated the impact of different inoculation doses and routes on these parameters.

## Materials and methods

### Challenge virus

The EHDV-8 strain SPA 2022/LCV_03 was kindly provided by the Spanish National Reference Laboratory for EHD and was used as a challenge strain in our experiment. Before receipt, the strain was passaged on KC cells (RRID: CVCL_RW99); afterward, it was further passaged twice and titrated on BHK-21 cells (RRID: CVCL_1914) in our laboratory (titre 10^6.2^ TCID_50_/mL).

### Animal trial

The animal experiment, which was conducted at Sciensano, took place in vector-proof BSL3 stables. All the animals were 6-month-old Holstein bulls that were confirmed to be free of bluetongue virus (BTV), EHDV, bovine viral diarrhoea virus and bovine herpesvirus-1. Upon arrival, they underwent a 5-day acclimatization period to minimize any transport-related stress effects on their health parameters [23]. Insecticides were used to treat the animals before challenge, and two black light traps were placed within the stables and monitored on a regulatory basis for the presence of *Culicoides*.

Twelve animals were randomly divided into four groups of three animals each. Three groups were challenged with EHDV-8: Group 1 (EHDV01-EHDV03) was inoculated intradermally with 1 mL (5 × 0.2 mL) of the virus stock, accounting for a final dose of 10^6.2^ TCID_50_/animal, which was considered the high dose; Group 2 (EHDV04-EHDV06) was inoculated subcutaneously with 1 mL of virus stock (10^6.2^ TCID_50_/animal; high dose); and Group 3 (EHDV07-EHDV09) was inoculated intradermally with 1 mL (5 × 0.2 mL) of 1/10 diluted virus stock, corresponding to 10^5.2^ TCID_50_/animal, which was considered the medium dose. The fourth group (Mock01‒Mock03) was injected intradermally (1 mL; 5 × 0.2 mL) and subcutaneously (1 mL) with MEM (Fisher Scientific, Belgium) and served as the mock-inoculated control group. All inoculations were administered in the neck region. Euthanasia was carried out 21 days post-inoculation (dpi).

### Clinical evaluation and sampling

All animals were clinically monitored daily from the acclimatization period until the end of the trial. The clinical evaluation was based on different parameters that were scored according to Table 1. Additionally, body temperature was measured daily and classified as normal if it was below 39.5 °C or as fever if it was at or above 39.5 °C.

**Table 1:** **Clinical scoring table**

Score	Nasal discharges	Breathing	General condition	Appetite	Conjunctivitis	Lesions in the mouth	Prescapular lymph nodes
0	Normal	Normal	Normal	Normal	Absent	None	Normal
1	Mild mucous	Increased respiration	Slight depression	Reduced	Present	Mild	Enlarged
2	Marked mucous	Abdominal breathing	Lethargy	No appetite		Severe	
3	Purulent	Gasping	Down				

Samples (EDTA blood, heparin blood, serum, nasal and buccal swabs) were collected at regular intervals throughout the trial. Sampling occurred once during the acclimatization period, on the day of inoculation (0 dpi), and at 3, 5, 7, 10, 13, 17 and 19 dpi. Additionally, skin biopsies were collected once a week during the post-inoculation period at 5, 10 and 17 dpi. At necropsy, 25 tissue and organ samples were collected from each animal challenged with EHDV-8. These samples were categorized into 5 groups: skin samples (skin at the inoculation site, normal skin, muzzle, nasal mucosa, mouth mucosa and coronary band), lymph nodes (inguinal ln, prescapular ln, submandibular ln, bronchial ln, mesenteric ln, parotid ln, iliac ln, mediastinal ln and tonsils), internal organs (lung, spleen, liver and kidney), muscles (heart and tongue) and brain samples (olfactory bulbus, cerebrum, cerebellum and brain stem).

### RNA extraction

RNA was extracted from EDTA blood samples using the QIAamp Viral RNA Kit (Qiagen, Antwerp, Belgium) and from nasal, buccal, and organ samples using the Indimag 48 extraction robot with the Indimag Pathogen Kit (INDICAL BIOSCIENCE GmbH, Leipzig, Germany), following the manufacturers’ protocols. Before RNA extraction, the swab samples were homogenized in 1 mL of PBS by vortexing, while the organ samples (~0.5 cm^3^) were homogenized with beads in 0.5 mL of PBS using a tissue lyser (2 cycles of 2 min at 25 Hz) and centrifuged at 10 000 rpm for 5 min.

### EHDV RNA detection by RT‒qPCR

A pan-EHDV RT‒qPCR assay targeting segment 9 was used [24] in a duplex reaction with GADPH as an endogenous control using the following primers and probes: GADPH_F (5’-TCACCATCTTCCAGGAGCGAG-3’), GADPH_R (5’AAGGTGCAGAGATGATGACCCTC-3’) and GADPH_P (HEX-CAAGTGGGGTGATGCTGGTGCTGAGTA-BHQ1).

Amplifications were performed via the AgPath-ID One-Step RT‒PCR Kit (Thermo Fisher) with the following reaction mixture: 12.5 μL of RT‒PCR Buffer (2X), 1 μL of RT‒PCR enzyme mixture (25X), 2 μL of primer/probe mixture, 4.5 μL of nuclease-free water and 5 μL of denatured RNA extract. The final concentrations of the primers and probes were 0.4 μM and 0.2 μM, respectively, for EHDV and 0.1 μM and 0.04 μM, respectively, for GADPH. RNA denaturation was performed by heating the RNA extracts at 95 °C for 3 min just before their addition to the reaction mixture. Amplification was performed under the following cycling conditions:

45 °C for 10 min, 95 °C for 10 min, followed by 45 cycles at 95 °C for 15 s and 60 °C for 45 s. Samples with Ct values ≤ 40 and exhibiting exponential amplification curves were considered positive. A Ct value < 35 for the endogenous control (GAPDH) was required for a sample to be valid.

### Virus isolation

The presence of infectious virus in the blood was evaluated by inoculating 1 mL of lysed blood on confluent monolayers of KC cells at 24 h after seeding in 25 cm^2^ flasks. After 1 h of incubation at room temperature, 9 mL of Schneider medium (supplemented with 10% FCS, 1 µg/mL fungizone and 20 µg/mL gentamycin) was added. The inoculated flasks were incubated at 28 °C for 7 days. After this period, the cells were frozen, and after thawing, the supernatants were analysed by RT‒PCR to assess virus replication by comparing the Ct values between day 0 and day 7. If no virus replication was detected after the first passage, subsequent passages up to a maximum of three were performed before classifying the sample as negative.

### EHDV ELISA

EHDV antibodies were detected using a commercial VP7 competition ELISA (ID SCREEN EHDV competition kit, Innovative Diagnostics, France) following the manufacturer’s protocol. The results are represented as a percentage of competition (S/N%), which was calculated using the following formula: (OD of the sample/OD of the negative control) × 100. The samples were classified as negative if the S/N% values were ≥ 40, doubtful if they were between > 30 and < 40, and positive if they were ≤ 30.

### Virus neutralization test (VNT)

VNT was conducted to evaluate the presence of neutralizing antibodies against the EHDV-8 strain SPA 2022/LCV_03. EHDV-8-positive control sera and negative bovine sera were included as positive and negative controls, respectively. Fifty microliters of twofold serially diluted serum from 1:10 to 1:1280 was incubated with 50 μL of virus dilution containing 100 TCID_50_ of the EHDV-8 strain for 1 h at 37 °C. After 1 h, 50 μL of BHK cell suspension (2 × 10^4^ cells) was added to each well. After a 3-day incubation period at 37 °C, the wells were evaluated under a light microscope for the presence of a cytopathic effect (CPE). The neutralizing antibody titre was determined as the reciprocal of the highest serum dilution that inhibited CPE in 50% of the wells.

### Interferon-gamma release assay

For each sampling time and animal, 1.5 mL of heparin blood was added to three wells of a 24-well plate. The wells were stimulated with 100 µL of EHDV-8 virus stock (10^6.2^ TCID_50_/mL), PBS as a negative control, or Pokeweed Mitogen (160 µg/mL) as a positive control. The plates were incubated overnight (16–24 h) at 37 °C with 5% CO_2_. Following incubation, the plates were centrifuged at 500 × *g* for 10 min, and 500 µL of the supernatant was collected and stored at −20 °C.

IFN-γ concentrations in the supernatants were measured using the ID Screen^®^ Ruminant IFN-γ ELISA according to the manufacturer’s protocol. A cut-off value of 30% was used to determine positivity. The OD values of the positive and negative controls were monitored to evaluate T-cell responsiveness and detect potential false-positive or false-negative results.

### Statistical analysis

Chi-square tests were performed to evaluate potentially significant differences in the number of fever days among the different EHDV-8 infection groups and to compare the number of positive biopsies per collection date. One-way ANOVA was used to assess differences in peak viremia and viral loads in organs between groups. Student’s *t* tests were applied to compare Ct values in the blood at different time points. The Kruskal‒Wallis test was used to compare neutralizing antibody response levels between the different EHDV-8-inoculated groups at specific time points. Statistical analyses were performed in GraphPad Prism 9, and differences were considered significant if *p* < 0.05.

## Results

### Clinical observations

**Figure 1:**
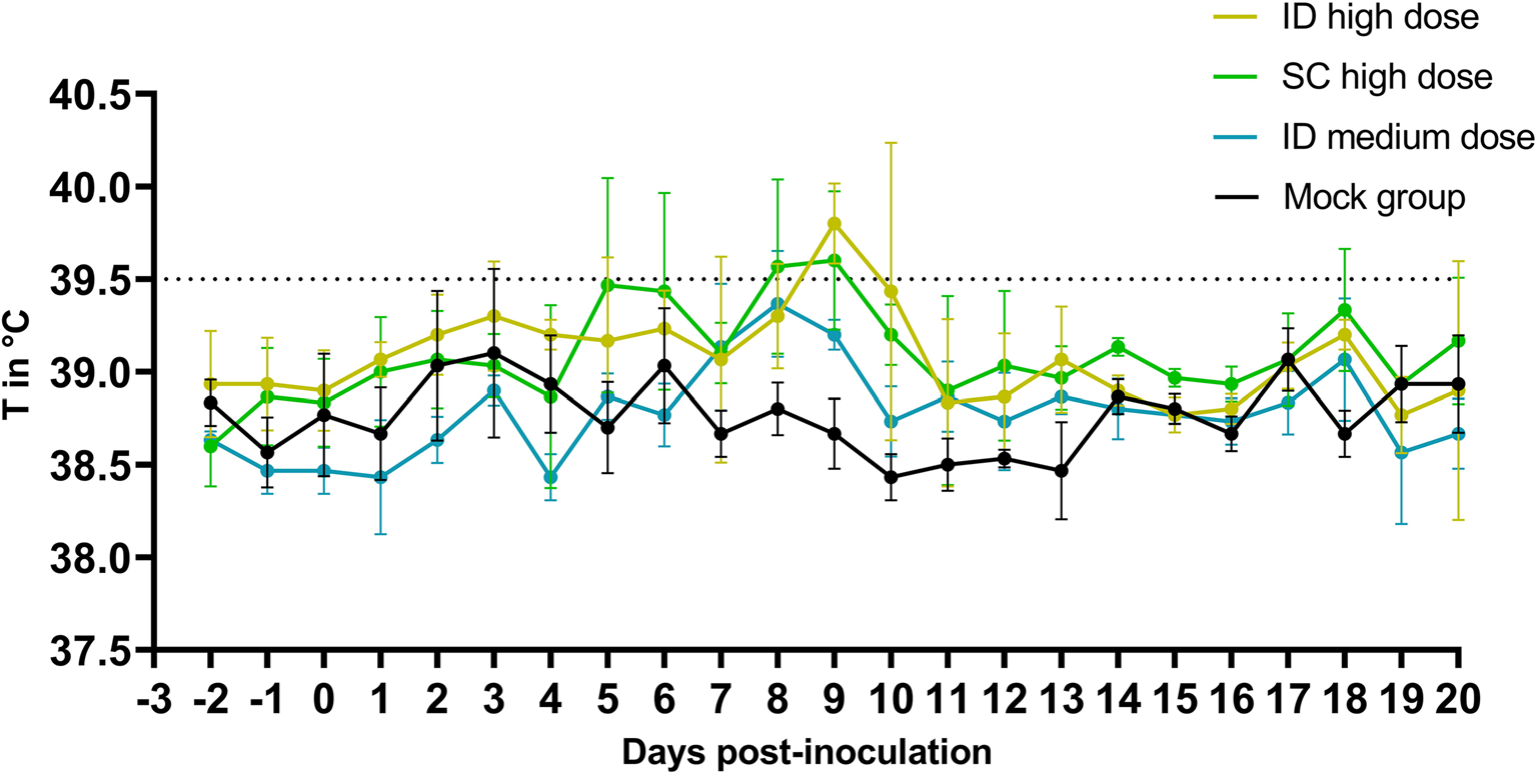
**Averaged body temperatures of the different inoculation groups.** Groups of three animals were inoculated intradermally (ID) or subcutaneously (SC) with a high dose (10^6.2^ TCID_50_/animal) or intradermally with a medium dose (10^5.2^ TCID_50_/animal). A negative control group, which was not inoculated, was included for comparison. Dotted line: fever cut-off; standard deviation is shown as error bars.

The animals in the intradermal (ID) high-dose and subcutaneous (SC) high-dose groups presented clear fever spikes, particularly at 8–9 dpi. Most animals (5 out of 6) in these groups exceeded the fever threshold of 39.5 °C, reaching maximum temperatures between 39.7 and 40.1 °C. The ID medium-dose group had a milder response, with only one animal having fever for 2 days (39.6 °C and 39.7 °C). The number of fever days between 5 and 10 dpi differed significantly between the groups (chi-square test, *p* < 0.05), with a total of 10 fever days observed in the ID high-dose group, 7 in the SC high-dose group, and 2 in the ID medium-dose group. The mock group consistently maintained body temperatures below the fever threshold of 39.5 °C (Figure 1). Except for fever, no other clinical signs were observed across the groups, except for one animal in the SC high-dose group (EHDV04). Starting at 10 dpi, this animal exhibited apathy, swollen lymph nodes (noted at 12 dpi), and conjunctivitis (observed at 15–16 dpi).

### Viremia

**Figure 2:**
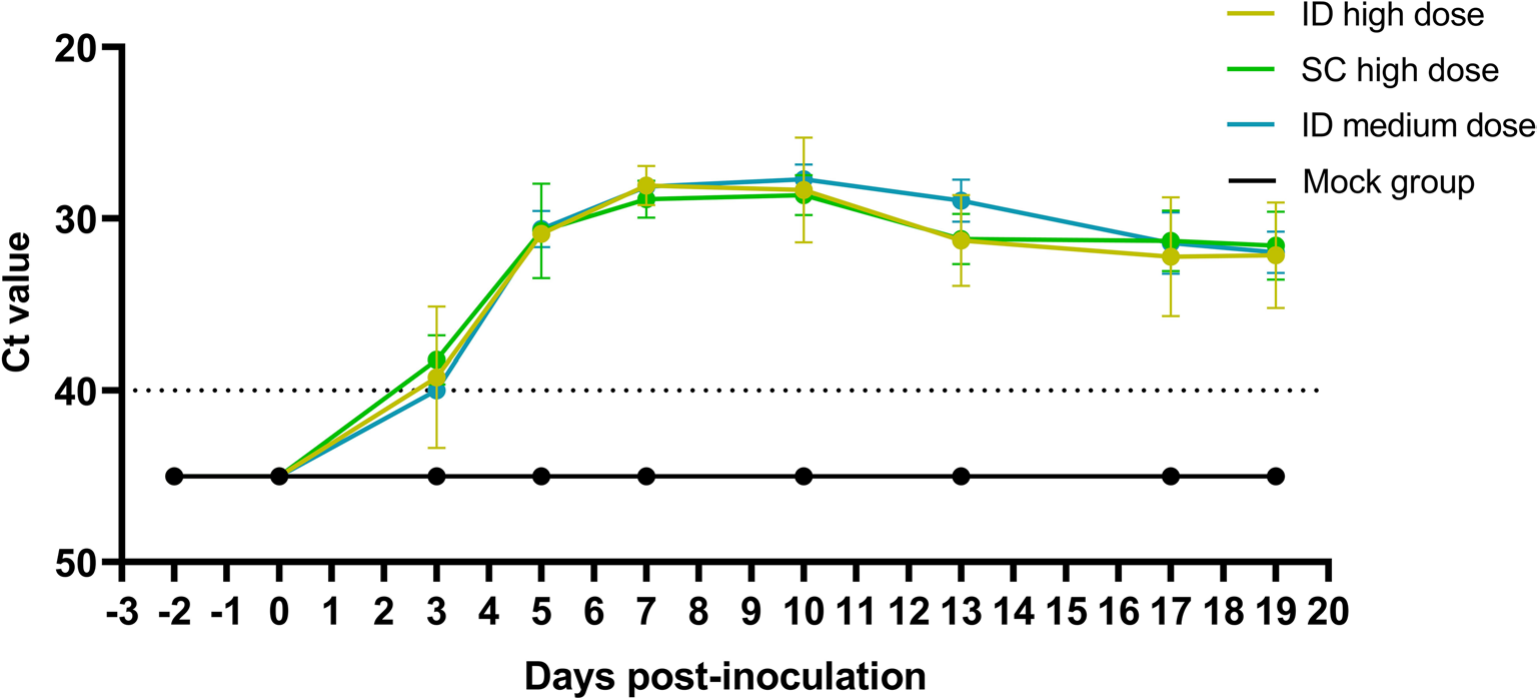
**Averaged Ct values in blood samples from the different inoculation groups**. Groups of three animals were inoculated intradermally (ID) or subcutaneously (SC) with a high dose (10^6.2^ TCID_50_/animal) or intradermally with a medium dose (10^5.2^ TCID_50_/animal). A negative control group, which was not inoculated, was included for comparison. Dotted line: cut-off; standard deviation is shown as error bars.

At 3 dpi, EHDV RNA was detected in the blood of all infected animals, with Ct values ranging from 36 to 40, except for one animal in the ID high-dose group, which first tested positive on day 5. Peak viremia occurred between days 7 and 10 across all infected groups, with Ct values ranging from 25 to 32. No significant differences in Ct values at peak viremia were found between the EHDV-8-inoculated groups (one-way ANOVA; *p* = 0.68). At 13 dpi, RNA levels in the blood were significantly lower than those at peak viremia (t test; *p* < 0.001) and further decreased by day 19 (t test; *p* < 0.05). The mock group consistently showed undetectable viremia (Ct > 40) throughout the trial (Figure 2).

In the SC high-dose group, the infectious virus was isolated from the blood of one animal at 3 dpi (Ct blood: 36.59), from all animals at 5, 7 and 10 dpi (Ct 27.18–33.77; 27.46–30.03; 27.05–29.69; respectively), and from one animal at 13 dpi (Ct 30.72). Except at 3 dpi, similar virus isolation results were obtained in the ID medium-dose group. The infectious virus was recovered from one animal at 5 dpi (Ct 29.24) and 13 dpi (Ct 27.74) and from all animals at 7 dpi (Ct 27.74–28.59) and 10 dpi (Ct 26.6–28.62). In contrast, in the ID high-dose group, the infectious virus was only isolated from the blood of all the animals at 7 dpi (Ct 26.51–28.92) and 10 dpi (Ct 25.79–32.60) but not at 3, 5 or 13 dpi.

### Virus detection in swabs and skin

Only a limited number of nasal and buccal swabs across the infected groups tested RT‒qPCR positive, with borderline positive Ct values between 37 and 40. The sole exception was one positive nasal swab (Ct 34.5) detected at 10 dpi in an animal from the ID medium-dose group. All swabs collected from the mock group were negative.

Viral RNA was detected in skin biopsies from 89% of the inoculated animals during at least one sampling. Interestingly, samples from the clinically affected animal (SC-high dose) consistently tested negative. At 5 dpi, biopsies from 4 out of 9 animals tested positive, with a mean Ct value of 40, significantly increasing to 8 out of 9 animals at 10 dpi (chi-square test: *p* < 0.05), with a mean Ct value of 37, indicating a significant increase (t test: *p* < 0.05) in viral load. By 17 dpi, only 3 out of 9 biopsies remained positive, with a mean Ct value of 37, indicating a significant decrease in the number of positive samples compared with that at 10 dpi (chi-square test: *p* < 0.05), although the viral load in the positive biopsies remained stable (mean Ct = 37; t test: *p* < 0.05). Table 2 presents the number of positive animals per group and the mean Ct values at each collection date.

**Table 2:** **Detection of EHDV RNA in skin biopsies from the different inoculation groups**

Group		5 dpi	10 dpi	17 dpi
ID high dose	# positive animals	1/3	3/3	1/3
	Mean Ct value	40.0	38.5	37.3
SC high dose	# positive animals	1/3	2/3	0/3
	Mean Ct value	40.0	36.4	45.0
ID medium dose	# positive animals	2/3	3/3	2/3
	Mean Ct value	40.0	36.6	37.6
Mock	# positive animals	0/3	0/3	0/3
	Mean Ct value	45.0	45.0	45.0

Groups of three animals were inoculated intradermally (ID) or subcutaneously (SC) with a high dose (10^6.2^ TCID_50_/animal) or intradermally with a medium dose (10^5.2^ TCID_50_/animal). A negative control group, which was not inoculated, was included for comparison. Number of positive animals per group and mean Ct values at each collection date.

### Gross pathology and viral RNA detection at necropsy

Gross lesions were observed only in the kidney of the clinically affected animal (EHDV04), which presented visible petechiae on its surface (Additional file 1). EHDV-8 was successfully isolated from this organ.

**Figure 3:**
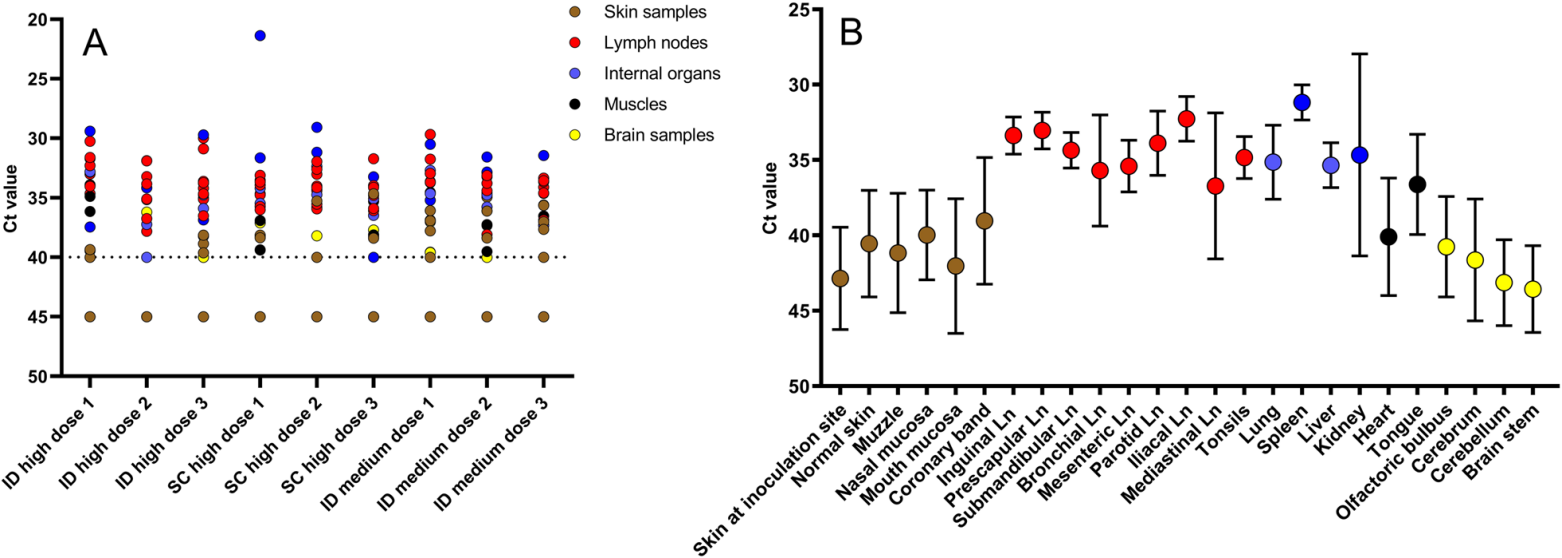
**Real-time PCR results of the organs collected at euthanasia. A** Ct values per individual animal; **B** average Ct values per organ across the different inoculation groups. Groups of three animals were inoculated intradermally (ID) or subcutaneously (SC) with a high dose (10^6.2^ TCID_50_/animal) or ID with a medium dose (10^5.2^ TCID_50_/animal). Dotted line: cut-off; standard deviation is shown as error bars.

The obtained Ct values for EHDV-8 ranged between 29 and 40 across the different organ/tissue samples. The only exception was the kidney of animal EHDV04, which had a Ct value of 20 (Figure 3A). The viral loads differed significantly between the different tissues (ANOVA: *p* < 0.001), with lower Ct values detected in the lymph nodes (mean Ct value: 33.99) and internal organs (mean Ct value: 34.13) than in the skin (mean Ct value: 37.92), brain (mean Ct value: 38.44), and muscle samples (mean Ct value: 36.46) (Figure 3B). A significant difference in viral load was also detected between the different internal organs (ANOVA: *p* < 0.001), whereby the spleen presented lower Ct values (mean Ct: 31.17) than did the lung, liver and kidney (mean Ct: 35.12; 35.34 and 35.08, respectively). The Ct value of the kidney from EHDV04 was excluded from this analysis, as it was identified as an outlier.

### Antibody response

**Figure 4:**
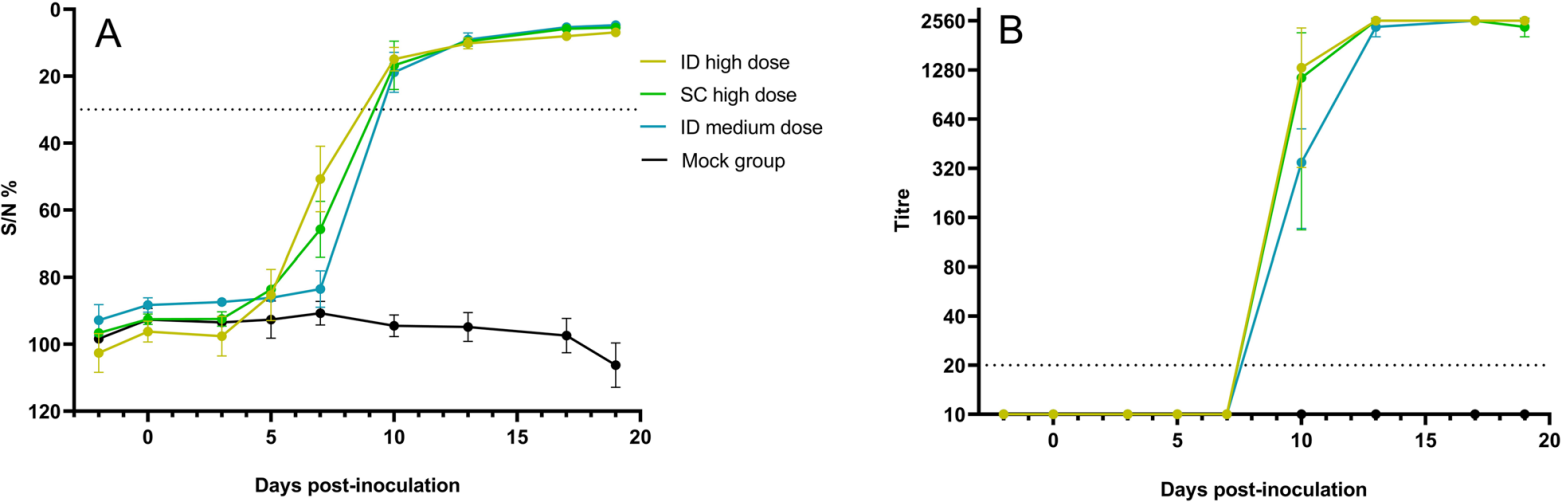
**Average S/N values (ELISA) (A) and neutralizing titres (VNT) (B) of the different inoculation groups.** Groups of three animals were inoculated intradermally (ID) or subcutaneously (SC) with a high dose (10^6.2^ TCID_50_/animal) or intradermally with a medium dose (10^5.2^ TCID_50_/animal). A negative control group, which was not inoculated, was included for comparison. Dotted line: positivity cut-off.

All infected calves became seropositive according to the ELISA at 10 dpi and remained positive until the end of the trial (Figure 4A). Virus neutralization tests further demonstrated that a neutralizing antibody response had developed in all infected animals from 10 dpi onwards, with high neutralizing antibody titres reaching 2560 or higher (the upper limit of detection) from 13 dpi in all the animals (Figure 4B). No difference in the timepoint of seroconversion was observed among the infected groups, and mock-infected animals remained seronegative. Although the VNT titres seemed lower in the ID medium group than in the other groups at 10 dpi, this difference was not significant (Kruskal‒Wallis test: *p* = 0.57).

### Interferon gamma release assay (IGRA) response

**Figure 5:**
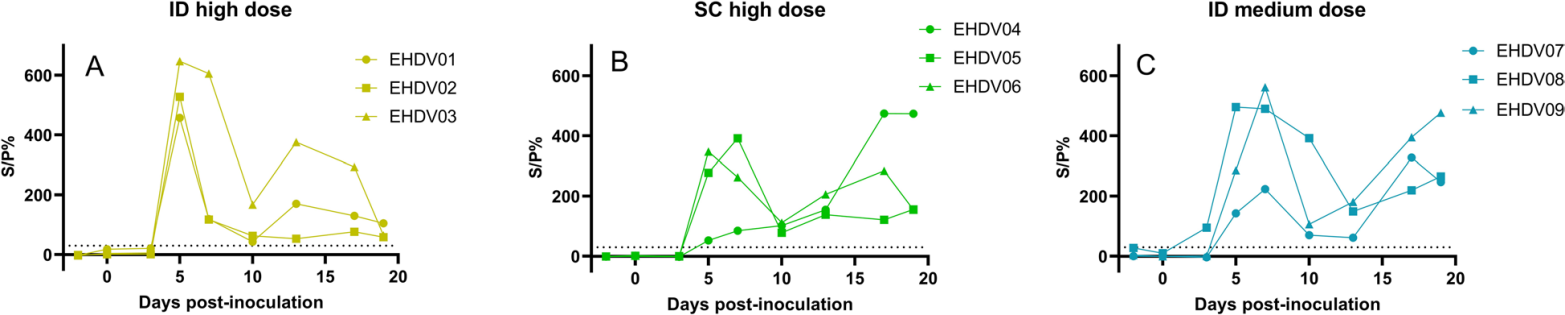
**IGRA results of the different inoculation groups.** Groups of three animals were inoculated intradermally (ID) or subcutaneously (SC) with a high dose (10^6.2^ TCID_50_/animal) (**A** and **B**) or ID with a medium dose (10^5.2^ TCID_50_/animal) (**C**). Dotted line: cut-off.

Following EHDV-8 inoculation, all infected groups presented a medium to strong IGRA response. Most animals showed a spike in cellular activity between 5 and 7 dpi, which was followed by a rapid decline to low positive results by 13 dpi. All the animals remained positive until the end of the trial, with occasional indications of a second, but lower, peak at approximately 19 dpi. Only EHDV04 from the SC high-dose group, which presented disease symptoms and high EHDV-8 replication in the kidney, displayed a divergent pattern. Specifically, no peak response was observed at 5 to 7 dpi, and a clear increase in IFN-gamma levels was detected only at 17 dpi (Figure 5). No cellular immune response was observed in the mock-infected animals throughout the study.

## Discussion

Although EHDV has circulated for 2 years in Italy, Spain, Portugal, and France, knowledge about its presence and persistence and induced immune responses in the most important hosts remains scarce. Currently, only limited epidemiological field data and few experimental infection studies focusing on EHDV-8 have been published. This poses a significant challenge for policymakers and hinders the development of efficient control and eradication strategies. Therefore, an experimental EHDV-8 infection study was performed in cattle.

To assess whether virus spread within the host and immune responses are influenced by the inoculation route and inoculation dose, we evaluated two administration methods. We compared the intradermal (ID) route with both high and medium doses to mimic natural *Culicoides* transmission [1] and a high-dose subcutaneous (SC) route, which has proven effective in previous studies [14]. Additionally, a placebo control group was included.

Fever was the only consistent clinical sign observed during the experiment. A comparison of fever duration revealed that, compared with those in the ID medium-dose group, the fever duration in both the ID high-dose and SC high-dose groups was prolonged. Only a single animal in the SC high-dose group presented additional clinical signs, such as apathy, swollen lymph nodes and conjunctivitis. Other recent studies using a European EHDV-8 isolate [22, 25] reported a broader range of clinical signs, including fever, lethargy, drooling, nasal lesions, and even mortality, in one calf. Field data from recent EHDV-8 outbreaks in Europe also indicate a wide range of clinical signs, including fever, nasal and oral lesions, conjunctivitis and occasional mortalities [15]. However, as discussed earlier, available field data remain limited. In contrast to the observations for the European EHDV-8 strain, no clinical symptoms were observed in cattle inoculated with an Australian EHDV-8 strain [21]. This finding is consistent with other experimental infection studies with various EHDV serotypes, where the more severe pathogenicity observed in field cases has not been replicated under controlled experimental conditions [26–28]. The difference in clinical outcome observed between different experimental trials may be related to genomic variation between different isolates, such as those described between the recent circulating EHDV-8 strain in Europe and North Africa and the Australian strain [17]. Additionally, variations in dose, inoculation route, or the number of passages on cells of the inoculum could have contributed to different outcomes in experimental studies [14].

Interestingly, petechiae were found on the kidney of the animal, which presented clinical symptoms in our study. The kidney contained a high viral load (Ct = 20) at euthanasia at 20 dpi, and infectious virus was still present. This is an interesting finding, as organ lesions were not observed in the EHDV-8 infection study by Spedicato et al. [22]. However, similar lesions in internal organs have been documented in red deer carcasses positive for EHDV-8 [16, 29]. Overall, the Ct values detected in the spleen and lungs in our study were comparable to those reported in the Italian study [22].

EHDV-8 RNA was detected in the blood of all calves, without significant differences between the groups concerning the onset, duration and height of viremia. Viral RNA was detected in the blood as early as 3 dpi, with a peak occurring between 7 and 10 dpi. The RNA levels gradually decreased afterwards but remained detectable until the end of the trial. Infectious EHDV-8 could only be detected until 13 dpi, suggesting that this is the timeframe during which infected animals could pass the virus to the *Culicoides* vectors and cause further transmission. This finding is in line with other experimental studies in which EHDV-8 was isolated up to 10–11 dpi in most infected calves, except for one calf from which live virus was isolated up to 21 dpi [22, 25]. As is known for other orbiviruses, such as Bluetongue [30], the RNAemia of EHDV-8 is longer than the period during which the infectious virus is present. EHDV-8 RNAemia lasted up to 30 and 78 dpi in recent studies by Spedicato et al. [22, 25]. Comparable trends have also been noted for other EHDV serotypes, such as EHDV-1, EHDV-2 and EHDV-7 [26, 28]. The prolonged detection of viral RNA in blood highlights its importance as a diagnostic sample for both early detection and identification of infections at later stages.

The RNA present in the skin was found to be shorter and weaker than the RNAemia. For example, at 17 dpi, RNA was still detected in the blood of all the animals, with a mean Ct of 31, whereas it was detected in only 33% of the skin biopsies, with a mean Ct value of 37. Only traces of the viral genome could be detected in the swabs, further supporting the knowledge that vector transmission is the primary transmission route of EHDV [31].

At euthanasia, the highest viral loads were present in internal organs such as the spleen and lymph nodes (e.g., prescapular lymph nodes), confirming that these are the most interesting samples to test in the case of an EHDV suspicion in dead animals. Skin, muscle and brain samples consistently contained only low EHDV RNA loads.

Like other orbiviruses and in line with recent reports on EHDV-8, cattle develop a fast and strong humoral immune response upon infection with EHDV-8. All infected animals had seroconverted by 10 dpi and presented high neutralizing antibody titres regardless of the administered dose or route, indicating that serological tests are valuable tools for monitoring virus circulation in cattle. However, once animals are vaccinated with the recently available vaccine on the market, these tests will no longer be able to distinguish between vaccinated and infected animals. Therefore, the development of DIVA tests will be crucial for future surveillance and control strategies. In general, little is known about the cellular immune response induced by orbiviruses. Our results indicate that a cell-mediated immunity (CMI) response was already induced by 5–7 dpi but declined rapidly afterwards. Only the clinical animal presented a delayed CMI response, which peaked at 17 dpi. This delay in the cellular response may have contributed to the onset of clinical disease, as an early and strong cellular immune response is critical for controlling viral replication and preventing disease [32]. Since only one animal exhibited clinical signs in this study, further research with a larger sample size is needed to validate these findings.

In our study, EHDV-8 infection in cattle was characterized by early onset and long-lasting viremia, rapid seroconversion by 10 dpi with high neutralizing antibody titres, and high viral loads in internal organs such as the lymph nodes and spleen. This finding is in line with the recent findings of Spedicato et al. [22] and confirmed the diagnostic relevance of lymph nodes and the spleen. In addition to the study performed by Spedicato et al. [22], our study compared two different inoculum doses and evaluated both the intradermal and subcutaneous inoculation routes. A wider range of organs was assessed to study viral distribution, and we examined the cellular host immune response against EHDV-8. These findings led to the conclusion that the inoculation dose and route had only a limited impact on viral persistence and the induced immune response. A potential link between clinical disease and a suppressed cellular immune response was also detected, although this needs further validation. The results of this study provide essential insights for policymakers to refine surveillance protocols, improve early detection strategies, and support evidence-based decisions on animal movement and trade restrictions during outbreaks. Additionally, the infection model established in this study offers a valuable tool for evaluating vaccine safety and efficacy. Nonetheless, further research with a larger sample size is needed to confirm these results and deepen our understanding of EHDV-8 infection dynamics. Moreover, studies in other susceptible hosts, such as sheep, are crucial for assessing their role in virus circulation and transmission.

## Supplementary Information

**Additional file 1. Kidney with petechiae from animal EHDV04 in the subcutaneous high-dose group (10**^**6.2**^ **TCID**_**50**_**/animal).** Contrast-enhanced image to improve visualization of petechiae. Original untreated photo available on request.

## Data Availability

All the data generated or analysed during this study are included in this published article.
