# Supplementary figures and images for: Influence of inoculation dose and route on EHDV-8 distribution and the induced immune response in experimentally infected cattle
Source: Vet Res. 2025 Nov 21;56:222. doi: 10.1186/s13567-025-01652-3 (PMC12639689; doi:10.1186/s13567-025-01652-3)

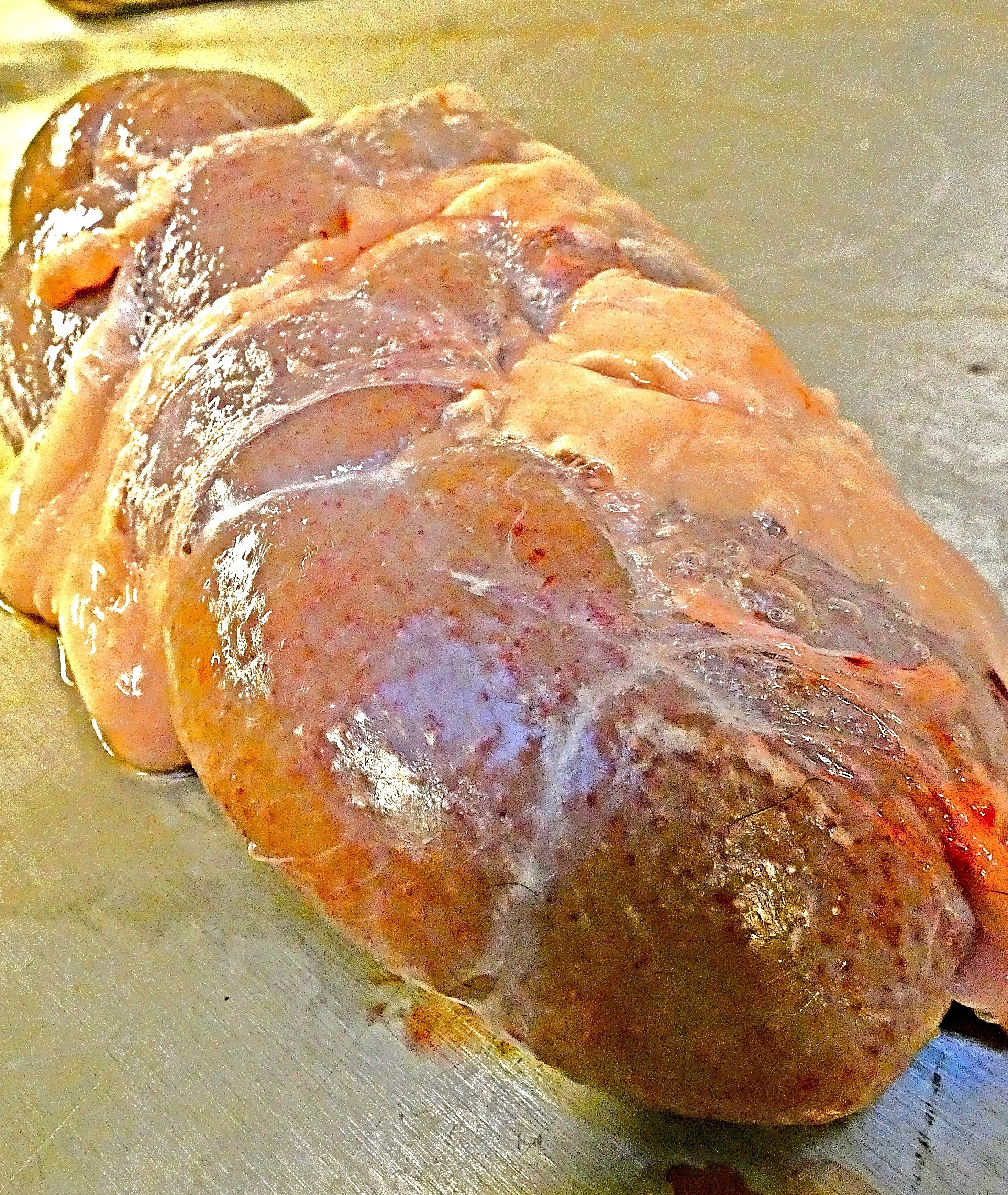

Supplement: Supplementary file 1 — Additional file 1. Kidney with petechiae from animal EHDV04 in the subcutaneous high-dose group (106.2 TCID50/animal). Contrast-enhanced image to improve visualization of petechiae. Original untreated photo available on request. [file 13567_2025_1652_MOESM1_ESM.jpg]
